# Echocardiographic Features of Cardiomyopathies: A Comprehensive Review

**DOI:** 10.1111/echo.70309

**Published:** 2025-10-09

**Authors:** Ghassan Hamdan Al‐Naami

**Affiliations:** ^1^ Family First Medical Clinic Edmonton Alberta Canada; ^2^ University of Alberta Edmonton Alberta Canada

**Keywords:** cardiac, cardiomyopathies, echocardiography, imaging, myocardial disease

## Abstract

Cardiomyopathies represent a heterogeneous group of myocardial diseases characterized by structural and functional abnormalities that can lead to heart failure, arrhythmias, and sudden cardiac death. Echocardiography remains the first‐line, non‐invasive imaging modality for the evaluation of cardiomyopathies, owing to its broad availability, safety profile, and diagnostic versatility. This review comprehensively outlines the echocardiographic features associated with the major morphofunctional subtypes of cardiomyopathy: dilated cardiomyopathy (DCM), hypertrophic cardiomyopathy (HCM), restrictive cardiomyopathy (RCM), arrhythmogenic right ventricular cardiomyopathy (ARVC), and left ventricular non‐compaction cardiomyopathy (LVNC). Emphasis is placed on both adult and pediatric populations, with attention to age‐specific diagnostic criteria and measurement standards. The article also includes structured tabular summaries to facilitate clinical interpretation and application across diverse patient settings. Through an integrated and standardized echocardiographic approach, this review aims to support accurate diagnosis, effective monitoring, and informed therapeutic decision‐making in the management of cardiomyopathies.

## Graphical abstract

Echocardiography remains a cornerstone in the diagnosis and characterization of cardiomyopathies, offering detailed structural and functional insights. This review outlines key echocardiographic features that guide the evaluation of dilated, hypertrophic, restrictive, and non‐compaction cardiomyopathies, enhancing clinical decision‐making.

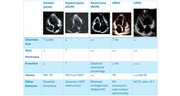

## 1 Introduction

**FIGURE 1:**
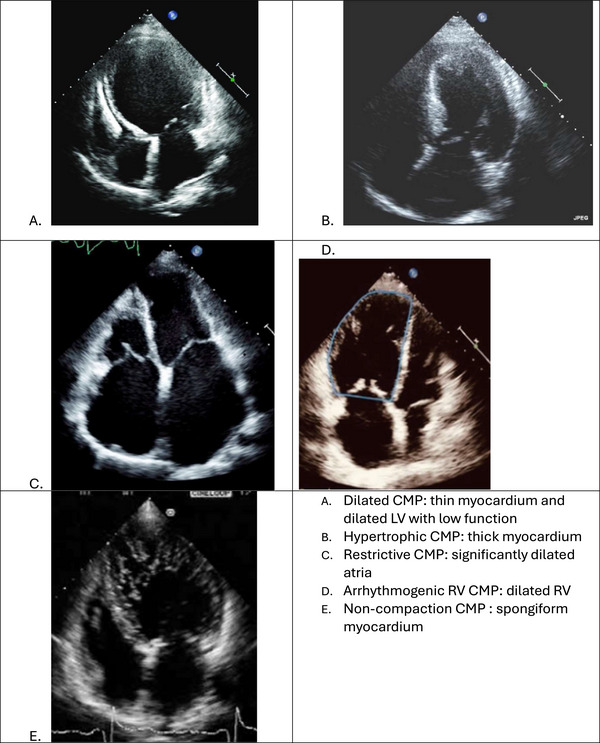
Major cardiomyopathies and their main echocardiographic features. (A) Dilated CMP: thin myocardium and dilated LV with low function. (B) Hypertrophic CMP: thick myocardium. (C) Restrictive CMP: significantly dilated atria. (D) Arrhythmogenic RV CMP: dilated RV. (E) Non‐compaction CMP: spongiform myocardium.

Cardiomyopathies represent a heterogeneous group of myocardial disorders that are classified based on morphofunctional characteristics into five major subtypes: dilated cardiomyopathy (DCM), hypertrophic cardiomyopathy (HCM), restrictive cardiomyopathy (RCM), arrhythmogenic cardiomyopathy (ACM), and left ventricular non‐compaction (LVNC). Figure 1 shows echocardiographic images for these types of cardiomyopathies. These conditions may be idiopathic or secondary to genetic, metabolic, infectious, or systemic causes, and they often lead to significant morbidity and mortality due to heart failure, arrhythmias, thromboembolic events, and sudden cardiac death. Early and accurate diagnosis is therefore paramount to optimizing patient outcomes and guiding appropriate therapeutic interventions.

Echocardiography remains the cornerstone of cardiomyopathy assessment due to its non‐invasive nature, widespread availability, and ability to provide real‐time structural, functional, and hemodynamic information (Table 1). It is indispensable in the initial diagnostic evaluation, risk stratification, serial monitoring, and follow‐up of both adult and pediatric patients with suspected or confirmed cardiomyopathies. Advanced echocardiographic modalities—including tissue Doppler imaging (TDI), speckle‐tracking echocardiography (STE), three‐dimensional (3D) echocardiography, and contrast‐enhanced studies—have further enhanced the precision of myocardial characterization and the detection of early or subtle phenotypic expressions.

**TABLE 1:** Key echocardiographic features of major cardiomyopathy types.

	Dilated (DCM)	Hypertrophic (HCM)	Restrictive (RCM)	ARVC	LVNC
Chamber size	↑ LV/RV	↓	↔	↑ RV	↓
Wall thickness	↓	↑	↔	↔	↔
Function	↓	↑	Diastolic restrictive physiology	↓ RV	↔‐↓
Valves	MR, TR	MR from SAM	↔	TR	↔‐MR/TR
Other features	Possible thrombus	Dynamic LVOT obstruction	Bilateral enlargement, dilated IVC	RV aneurysm, wall motion abnormality	NC/C ratio >2:1

Among these, global longitudinal strain (GLS) has emerged as a highly sensitive and reproducible echocardiographic parameter for the early detection of myocardial dysfunction, often preceding changes in left ventricular ejection fraction (LVEF). Unlike traditional volumetric indices, GLS assesses myocardial deformation by quantifying the percentage of longitudinal fiber shortening, particularly in the subendocardial layer, which is most vulnerable to early damage across cardiomyopathies [1, 2, 3, 4]. In **dilated cardiomyopathy (DCM)**, reduced GLS can identify subclinical systolic dysfunction before overt decline in LVEF, aiding in early diagnosis and monitoring therapeutic response. In **hypertrophic cardiomyopathy (HCM)**, GLS is typically reduced in hypertrophied myocardial segments despite preserved or hyperdynamic LVEF, and global impairment correlates with increased risk of heart failure and arrhythmias. In **restrictive cardiomyopathy (RCM)**, GLS may reveal global or regional dysfunction not apparent on conventional imaging, and in **cardiac amyloidosis**, the hallmark finding of apical sparing (“cherry‐on‐top” pattern) provides diagnostic specificity. In **arrhythmogenic right ventricular cardiomyopathy (ARVC)**, reduced longitudinal strain in the RV free wall may offer additional diagnostic value, especially when traditional measures are equivocal. For **left ventricular non‐compaction (LVNC)**, GLS abnormalities reflect impaired contractile function in non‐compacted segments and may predict clinical deterioration. The routine incorporation of GLS into echocardiographic assessment improves diagnostic precision, supports early risk stratification, and enhances prognostic evaluation across a broad spectrum of cardiomyopathies.

In parallel, the application of **artificial intelligence (AI)** and **machine learning (ML)** in cardiovascular imaging is rapidly expanding, offering promising new tools for the detection, classification, and risk stratification of cardiomyopathies. These technologies enable automated analysis of complex echocardiographic data, including feature extraction from strain patterns, myocardial texture, and motion dynamics, with the potential to reduce interobserver variability and improve diagnostic consistency. Additionally, **multimodal imaging fusion**—which integrates echocardiographic, cardiac MRI, CT, and even genetic or electrocardiographic data—enhances the ability to comprehensively characterize myocardial structure and function. In particular, AI‐assisted platforms may assist in distinguishing phenotypically overlapping conditions, such as differentiating hypertrophic cardiomyopathy from athlete's heart or early ARVC from normal variants. While these innovations hold considerable promise for improving diagnostic accuracy and workflow efficiency, they are currently in various stages of clinical validation. Rigorous prospective studies, regulatory approval, and integration into standardized imaging protocols are needed before widespread adoption in routine cardiomyopathy assessment.

Despite the widespread use of echocardiography, the interpretation of findings can vary significantly depending on patient age, body habitus, and institutional practices. As such, the application of standardized measurement protocols and age‐ and size‐specific normative data is critical to ensure diagnostic consistency and clinical reliability across different populations. In pediatric cardiology, where normal cardiac dimensions vary considerably with growth and development, the role of normative *z*‐scores and reference values becomes particularly important.

This review aims to synthesize key echocardiographic findings, measurement techniques, and diagnostic thresholds associated with each cardiomyopathy subtype. It emphasizes the role of standardized imaging approaches, highlights the nuances in pediatric versus adult assessments, and underscores the importance of integrating echocardiographic parameters into comprehensive clinical decision‐making frameworks.

**Pediatric considerations**: Pediatric measurements rely on *z*‐scores for any volumetric measurements. This supports the diagnosis of various cardiac disorders especially cardiomyopathies which relies much on accurate measurements.

Here are several reliable and commonly used **online *z*‐score calculators for pediatric echocardiography** (Table 2). These tools are based on validated datasets and are widely used in clinical practice to assess cardiac chamber size, wall thickness, and function in children [4, 5, 6, 7]:

1. Boston Children's Hospital *Z*‐Score Calculator
  - **Website**: https://zscore.chboston.org
  - **Features**:
    - Calculates *z*‐scores for multiple cardiac structures including LVEDD, LV mass, RV dimensions, aorta, and valves.
    - Based on Pettersen et al. (2008) [5] normative data.
    - Easy BSA calculation using height and weight.
    - Printable results and graphical output.
  - **Source Reference**: Pettersen et al. JASE 2008.
2. Pediatric *Z*‐Score Calculator (ParameterZ)
  - **Website**: https://parameterz.blogspot.com
  - **Features**:
    - Covers LV and RV size, wall thickness, and valve annuli.
    - Indexed to BSA, with multiple reference datasets.
    - Includes tools for both 2D and M‐mode parameters.
  - **Note**: Developed by a pediatric cardiologist; often used in academic settings.
3. *Z*‐Score Calculator by Children's Heart Center—Nevada
  - **Website**: https://www.pedz.de
  - **Features**:
    - Supports a wide range of z‐score calculations for pediatric echo.
    - Provides tables and percentile curves.
    - Includes calculators for both dimensions and functional parameters.
4. Congenital Heart Disease *Z*‐Scores—University of Michigan
  - **Website**: https://med.umich.edu/mott/pdf/zscore.pdf (PDF)
  - **Features**:
    - Manual *z*‐score lookup tables for a variety of cardiac measurements.
    - Good for reference or use in offline settings.

**TABLE 2:** Key reference studies for pediatric echocardiographic *z*‐score calculators.

Authors and source	Title	Journal/Year	Notes
Pettersen MD, Du W, Skeens ME, Humes RA	Regression Equations for Calculation of *Z* Scores of Cardiac Structures in a Large Cohort of Healthy Children	*J Am Soc Echocardiogr*, 2008; 21(8):922–934	Basis of the **Boston Children's *Z***‐score calculator; most widely used reference dataset.
Lopez L, Colan SD, Frommelt PC, et al.	Recommendations for Quantification Methods During the Performance of a Pediatric Echocardiogram	*J Am Soc Echocardiogr*, 2010; 23(5):465–495	ASE‐endorsed guidelines; widely followed in tools like **Parameter *Z*** and institutional protocols.
Foster BJ, Colan SD, Cheung YH, et al.	Pediatric Normative Data for Echocardiographic Indices: The Pediatric Heart Network Study	*Circ Cardiovasc Imaging*, 2010; 3(1):65–76	Robust multicenter normative data for pediatric cardiac function and dimensions.
Colan SD, Parness IA, Spevak PJ, Sanders SP	Developmental Modulation of Myocardial Mechanics: Age‐ and Growth‐Related Alterations in Afterload and Contractility	*J Am Coll Cardiol*, 1992; 19(3):619–629	Seminal work on scaling of LV dimensions; foundation for later normative modeling.

**Key Tips for Use**:

- Always input accurate height and weight to compute BSA reliably.
- Ensure the measurement method (e.g., PLAX vs. apical view) matches the dataset used by the calculator.
- Use consistent reference datasets when comparing values over time (e.g., for serial studies).
- Be aware of the growth charts and percentiles used in your local population, as some norms may differ slightly based on geography.

## 2 Echocardiographic Overview of Cardiomyopathy Subtypes

### 2.1 Dilated Cardiomyopathy (DCM)

Echocardiography plays a pivotal role in the diagnosis, evaluation, and management of **dilated cardiomyopathy (DCM)**, a condition characterized by **ventricular chamber enlargement** and **systolic dysfunction** in the absence of abnormal loading conditions or significant coronary artery disease. In DCM, echocardiography typically reveals a **dilated left ventricle** with **globular geometry**, **thinned walls**, and **reduced left ventricular ejection fraction (LVEF)**—often below 40%. The mitral valve may exhibit **functional regurgitation** due to annular dilation and papillary muscle displacement. Left atrial enlargement is common due to chronic volume overload. M‐mode and 2D imaging confirm chamber dilation and wall motion abnormalities, while **Doppler techniques** assess diastolic filling patterns, which may range from impaired relaxation to restrictive physiology in advanced disease. **Tissue Doppler imaging (TDI)** and **strain imaging** offer insights into subtle myocardial dysfunction and can detect early myocardial impairment even when ejection fraction is preserved. Right ventricular involvement, frequently seen in advanced cases, is assessed by TAPSE (tricuspid annular plane systolic excursion) and RV fractional area change. Echocardiography also evaluates for complications such as **intracavitary thrombus**, especially in the apex, which poses a risk for systemic embolism. Serial echocardiograms are essential in monitoring disease progression, response to therapy (e.g., with beta‐blockers, ACE inhibitors, or CRT), and candidacy for **advanced interventions** like implantable defibrillators or **cardiac transplantation**. Overall, echocardiography remains an indispensable, non‐invasive, and repeatable tool in the comprehensive care of patients with dilated cardiomyopathy [8, 9, 10, 11, 12].

DCM is characterized by ventricular chamber enlargement with impaired systolic function.

**FIGURE 2:**
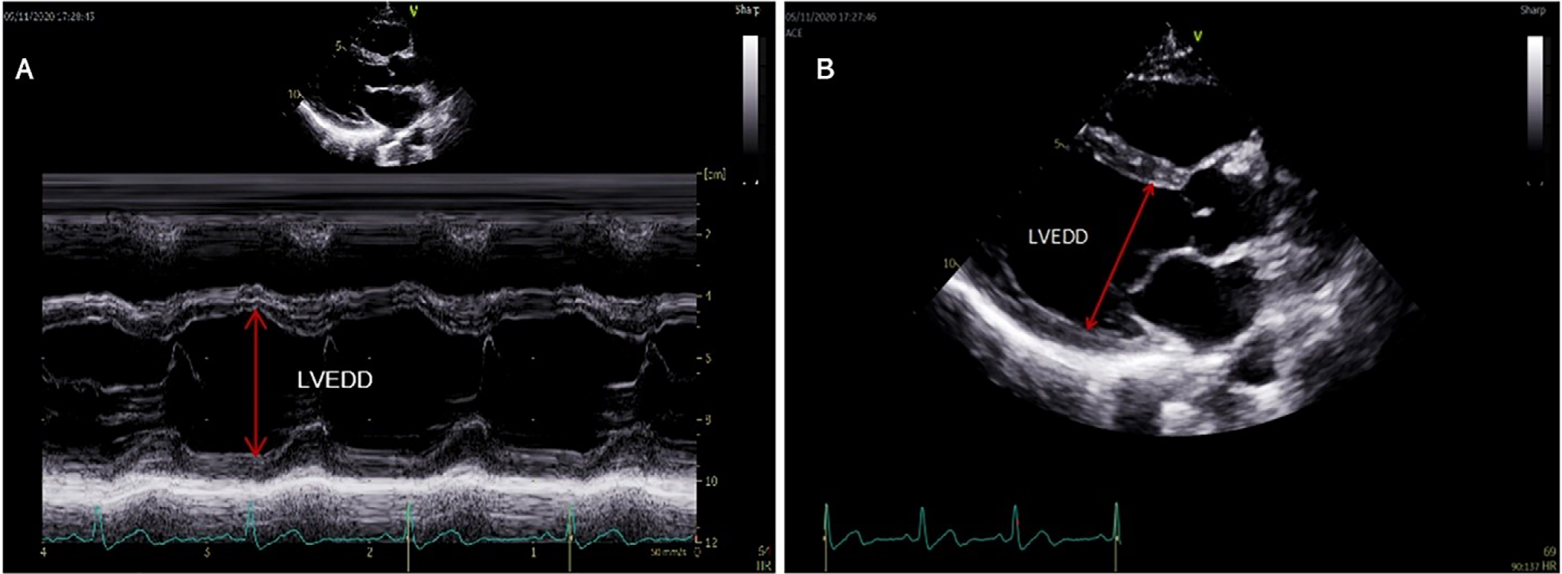
Measurement of left ventricular end‐diastolic diameter (LVEDD). (A) M‐mode echocardiogram showing LVEDD as the maximal internal dimension of the left ventricle during diastole. (B) 2D parasternal long‐axis view demonstrating the same measurement with directional annotation.

**Quantitative Assessment of LV Enlargement in Adults**:

- **LV End‐Diastolic Diameter (LVEDD)**: >58 mm (men), >52 mm (women)
  LVEDD is a key echocardiographic measurement used to assess left ventricular size and function. It represents the internal diameter of the left ventricle at the end of diastole, when the chamber is at its maximum filling. LVEDD is a critical parameter in evaluating volume status, ventricular remodeling, and early signs of dilated cardiomyopathy. It can be measured using 2D image or M‐mode of the left ventricle in parasternal long or short axis views (Figure 2). Normal reference values vary with age, sex, and body size, and deviations from these ranges can indicate pathological dilation or hypertrophy (Table 3). Accurate assessment of LVEDD is essential for diagnosing and monitoring various cardiac conditions especially in pediatric and adult populations with suspected myocardial disease like DCM (Table 4).
- **LV End‐Diastolic Volume Index (LVEDVi)**: >74 mL/m^2^ (men), >61 mL/m^2^ (women)
  LVEDVi **is a** measurement of the **volume of blood in the left ventricle at the end of diastole**, indexed to body surface area (BSA), and is typically expressed in **mL/m^2^**. It is a crucial echocardiographic and MRI parameter used to assess **left ventricular dilation and remodeling**, especially in the context of **Dilated Cardiomyopathy (DCM)**.

**TABLE 3:** Reference values for LV and RV (adults).

Parameter	Normal range (men)	Normal range (women)	Notes
LVEDD	42–58 mm	38–52 mm	PLAX view
LVEDVi	49–74 mL/m^2^	41–61 mL/m^2^	Biplane Simpson
LVMi	49–115 g/m^2^	43–95 g/m^2^	LV hypertrophy
RWT	<0.42	<0.42	Remodeling classification
RVD1	≤41 mm	N/A	Apical 4C view
RVEDAi	≤20 cm^2^/m^2^	≤20 cm^2^/m^2^	RV size measure
TAPSE	≥17 mm	≥17 mm	RV systolic function

**TABLE 4:** Comparative echocardiographic features of dilated CMP in adults versus children.

Feature	Adults	Children
LVEDD	>58 mm (men), >52 mm (women)	*z*‐score > +2
LVEDVi	>74 mL/m^2^ (men), >61 mL/m^2^ (women)	*z*‐score > +2
LV Mass Index	>115 g/m^2^ (men), >95 g/m^2^ (women)	*z*‐score > +2
RVD1	>41 mm	*z*‐score > +2
RVEDAi	>20 cm^2^/m^2^	*z*‐score > +2
TAPSE/FAC	Adult cutoffs	Pediatric norms

In DCM, the left ventricle becomes **enlarged and weakened**, leading to a **significantly elevated LVEDVi**. This reflects increased preload and poor systolic function due to **ventricular dilatation**.

It can be measured using:

**Cardiac MRI** is the gold standard for LV volume measurement.

Or simply, **2D or 3D echocardiography** can estimate LVEDVi using Simpson's biplane method, though less precise.

- LV **Mass Index**: >115 g/m^2^ (men), >95 g/m^2^ (women)
**LV Mass Index (LVMI)** quantifies the mass of the left ventricle (LV) relative to body surface area (BSA), expressed in **g/m^2^**. It reflects **myocardial hypertrophy or remodeling** and is an essential echocardiographic marker in many forms of cardiomyopathy, including **DCM**.
  The **LV chamber is dilated** and the wall may be **thinned**, but in many cases the **total LV mass increases** due to **eccentric hypertrophy** (myocyte elongation and fibrosis).
- LVMI is often **elevated**, especially in moderate to advanced stages of disease, reflecting **eccentric hypertrophy** (dilation + increased mass).
  Elevated LVMI is associated with:
  - Higher risk of arrhythmias
  - Progressive systolic dysfunction
  - Poorer prognosis in DCM patients
- **Relative Wall Thickness (RWT)**: <0.42 suggests eccentric remodeling
  It evaluates left ventricular geometry. It is calculated as twice the posterior wall thickness divided by the left ventricular end‐diastolic diameter (2 × PWT/LVEDD). RWT helps differentiate patterns of left ventricular remodeling, distinguishing between concentric and eccentric hypertrophy. An increased RWT suggests concentric remodeling or hypertrophy, often seen in pressure overload conditions such as hypertension, while a normal or reduced RWT may indicate volume overload or dilated cardiomyopathy.

**FIGURE 3:**
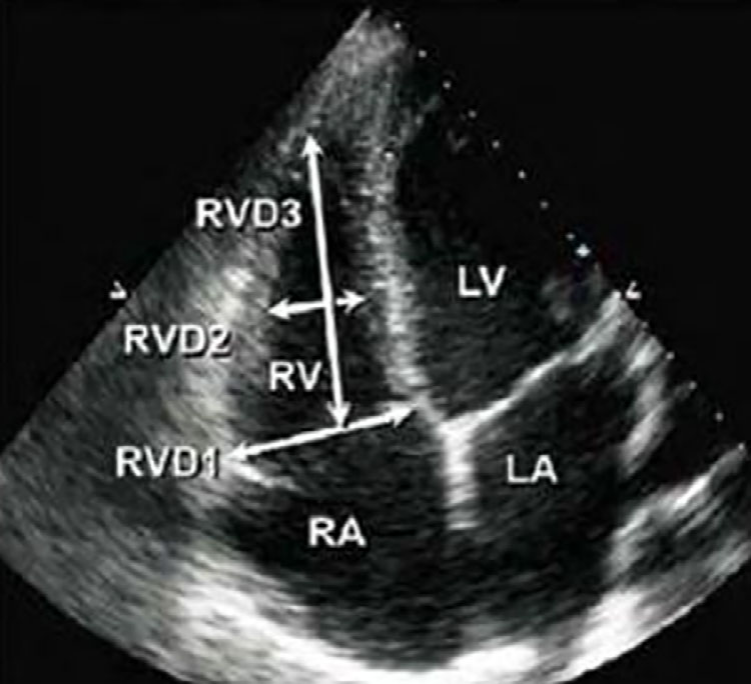
RV diameters used to quantify the RV size and remodeling conditions.

**Quantitative Assessment of RV Enlargement in Adults**:

- **RV Basal Diameter (RVD1)**: >41 mm
  The Right Ventricular Basal Diameter (RVD1) is an essential echocardiographic measurement that reflects the transverse dimension of the right ventricle at its widest point near the base, typically measured in the apical four‐chamber view. It provides a reliable estimate of right ventricular size and is commonly used in the assessment of right ventricular dilation. An increased RVD1 may indicate conditions such as pulmonary hypertension, right heart failure, or congenital heart disease.
- **RV Mid‐Cavity Diameter (RVD2)**: >35 mm
  It is measured at the mid‐level of the right ventricle, approximately halfway between the base and the apex, in the apical four‐chamber view. It provides valuable information about the mid‐portion of the right ventricular chamber and is used to assess right ventricular size and remodeling. Enlargement of RVD2 may indicate right ventricular dilation due to pressure or volume overload, as seen in pulmonary hypertension, congenital heart disease, or cardiomyopathies.
- **RV Length (RVD3)**: >86 mm
  Also known as the longitudinal dimension, is measured from the tricuspid annulus to the apex of the right ventricle in the apical four‐chamber view. It reflects the longitudinal size of the right ventricle and complements other measurements such as RVD1 and RVD2 in assessing overall right ventricular dimensions. An increased RVD3 may indicate right ventricular dilation due to conditions like pulmonary hypertension, right‐sided volume overload, or cardiomyopathy.
  Figure 3 demonstrates these three diameters.
- **RVEDA Index**: >20 cm^2^/m^2^
  The Right Ventricular End‐Diastolic Area (RVEDA) Index is an echocardiographic parameter that represents the area of the right ventricle at end‐diastole, indexed to body surface area to allow for size‐related comparisons across individuals. Measured in the apical four‐chamber view, the RVEDA Index is used to assess right ventricular size and enlargement. An increased RVEDA Index is indicative of right ventricular dilation, commonly associated with pulmonary hypertension, right heart failure, or congenital heart defects. It is a valuable tool for evaluating right ventricular remodeling and guiding clinical management in both pediatric and adult patients.
- **RV FAC**: <35% indicates systolic dysfunction
  Right Ventricular Fractional Area Change (RV FAC) is a widely used echocardiographic parameter that quantifies right ventricular systolic function. It is calculated as the percentage change in the right ventricular area from end‐diastole to end‐systole using the formula: [(RVEDA—RVESA)/RVEDA] × 100, where RVEDA is the end‐diastolic area and RVESA is the end‐systolic area. Measured in the apical four‐chamber view, RV FAC reflects global right ventricular contractility. A reduced RV FAC (<35%) suggests impaired systolic function and may be seen in conditions such as pulmonary hypertension, right heart failure, or cardiomyopathies.

### 2.2 Hypertrophic Cardiomyopathy (HCM)

**FIGURE 4:**
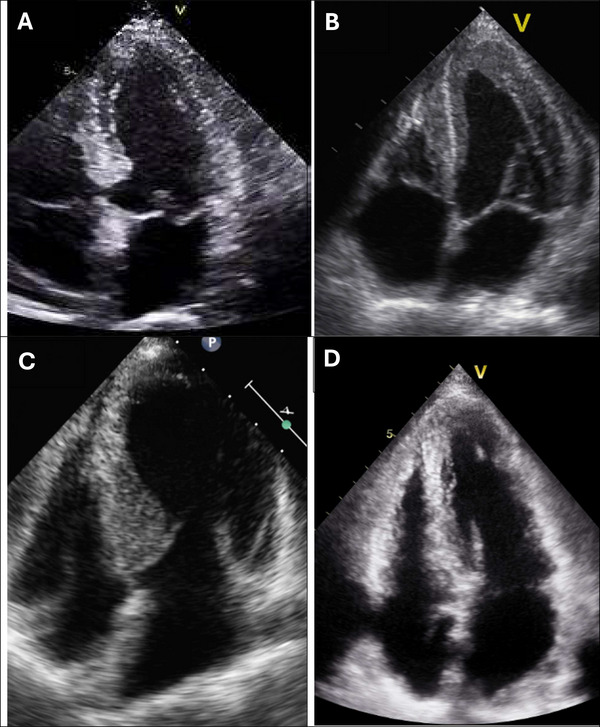
Echocardiographic variants of HCM. (A) Asymmetric septal hypertrophy with a reverse curvature of the interventricular septum. (B) Symmetric septal hypertrophy with a neutral septal contour. (C) Sigmoid‐shaped septal hypertrophy localized to the basal portion of the septum. (D) Apical hypertrophy limited to the apex, with normal wall thickness in the basal segments.

**FIGURE 5:**
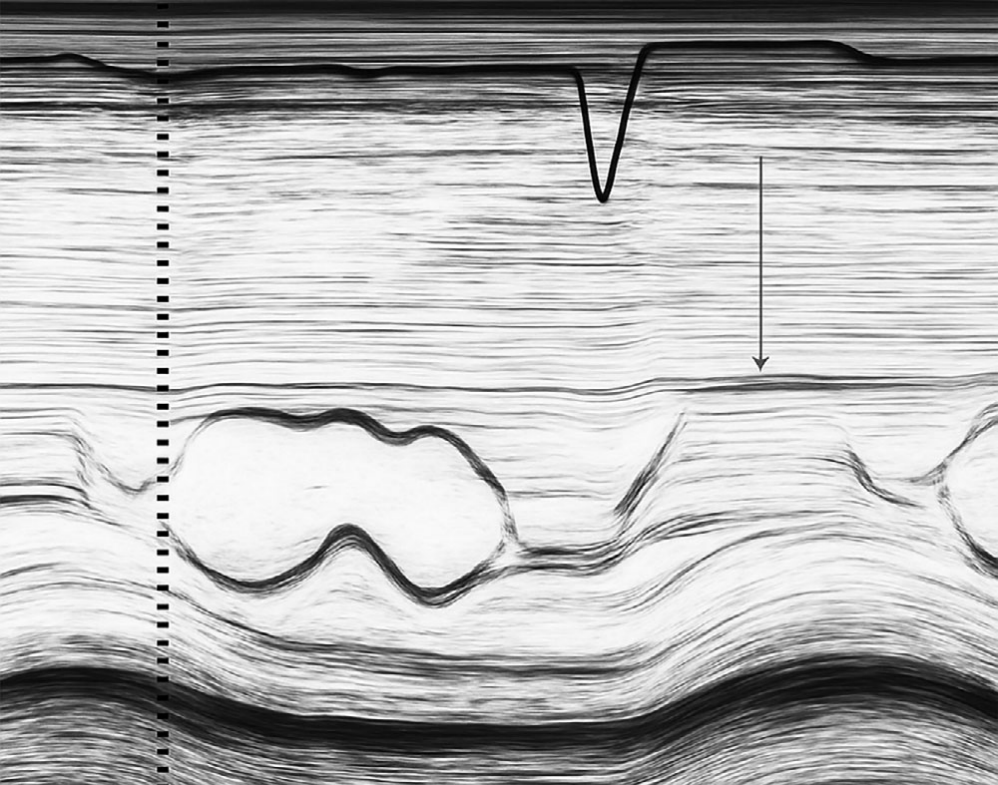
M‐mode echocardiogram demonstrating systolic anterior motion (SAM) of the mitral valve. The image captures the characteristic anterior displacement of the mitral valve leaflet during systole, indicative of hypertrophic obstructive cardiomyopathy (HOCM). The arrow marks the point of maximal anterior motion toward the interventricular septum, contributing to dynamic left ventricular outflow tract (LVOT) obstruction.

**FIGURE 6:**
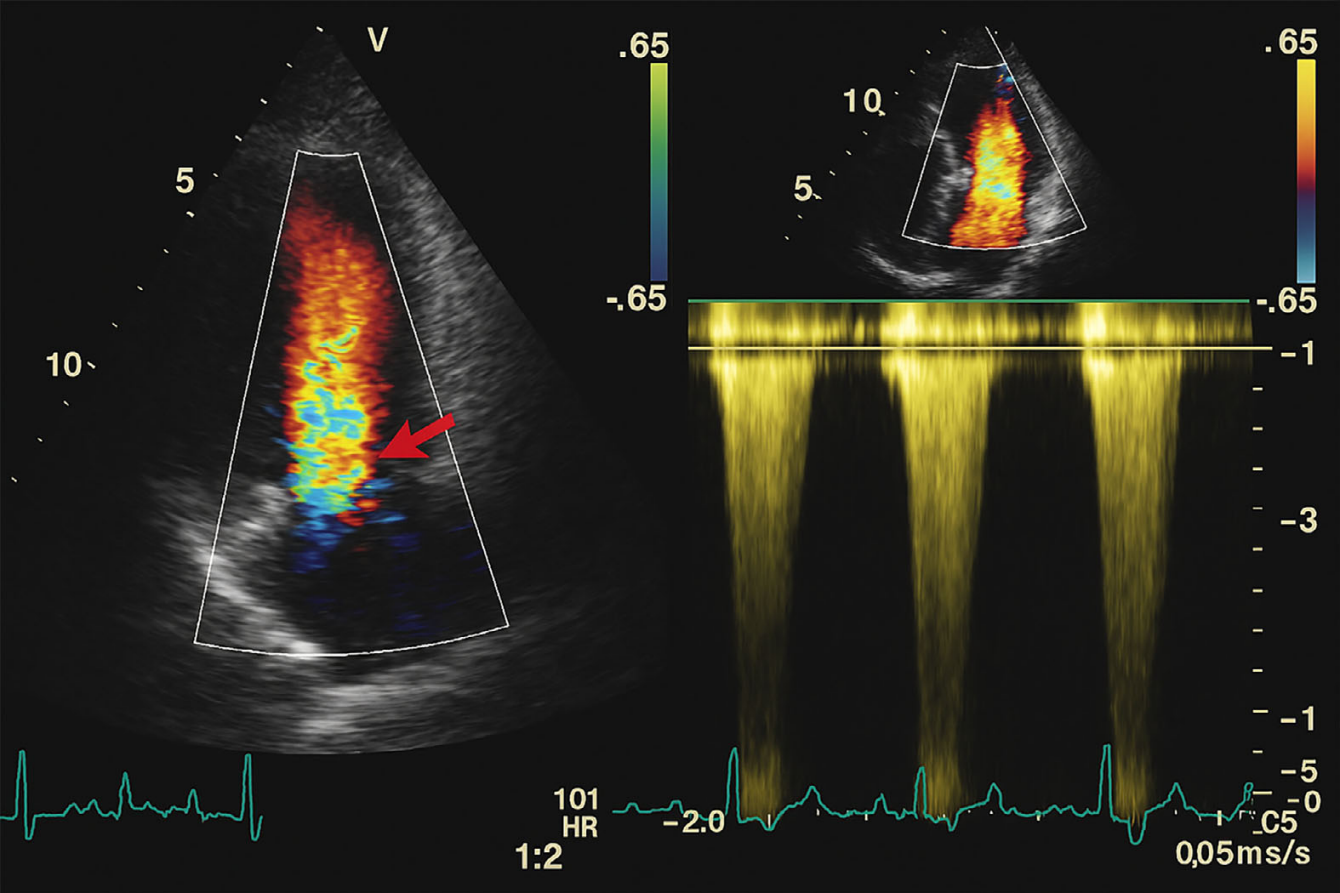
Doppler echocardiographic image demonstrating dynamic left ventricular outflow tract (LVOT) obstruction highlighting a turbulent jet (red arrow) consistent with systolic anterior motion (SAM) of the mitral valve and LVOT obstruction.

Echocardiography is the cornerstone imaging modality in the diagnosis, phenotypic assessment, and management of **hypertrophic cardiomyopathy (HCM)**, a genetically determined myocardial disease characterized by **left ventricular hypertrophy** (LVH) in the absence of abnormal loading conditions such as hypertension or aortic stenosis. Conventional two‐dimensional transthoracic echocardiography allows for the assessment of **asymmetric septal hypertrophy**, which is the most common morphological variant, as well as other patterns including concentric, apical, or mid‐ventricular hypertrophy (Figure 4). Left ventricular wall thickness exceeding 15 mm in one or more segments is diagnostic, with the interventricular septum typically being most affected. M‐mode imaging may demonstrate systolic anterior motion (SAM) of the mitral valve, which contributes to **left ventricular outflow tract (LVOT) obstruction**—a dynamic feature present in approximately 70% of patients either at rest or with provocation (Figure 5). Doppler echocardiography quantifies the **LVOT gradient**, which is a critical determinant of symptoms and treatment decisions; a resting or provocable gradient ≥30 mmHg is considered significant, while ≥50 mmHg often warrants septal reduction therapy (Figure 6). Color Doppler also aids in assessing **mitral regurgitation** secondary to SAM. Advanced echocardiographic techniques such as **tissue Doppler imaging (TDI)** and **speckle‐tracking echocardiography (STE)** can detect subclinical myocardial dysfunction, even when ejection fraction appears preserved, and may provide prognostic information. Left atrial enlargement, reduced left ventricular cavity size, and abnormal diastolic filling patterns are also common findings. Transesophageal echocardiography (TEE) and contrast echocardiography may be used in specific clinical scenarios, especially when image quality is suboptimal. Furthermore, serial echocardiographic assessments are crucial for risk stratification, particularly in evaluating the evolution of hypertrophy, obstruction, and detection of apical aneurysms or thrombi. In patients with ambiguous morphology or discordant findings, **cardiac magnetic resonance imaging (CMR)** serves as a valuable adjunct. Nonetheless, echocardiography remains the first‐line, accessible, and dynamic tool for diagnosing HCM, guiding clinical decisions, and monitoring therapy effectiveness, including the impact of pharmacologic agents and the need for surgical or interventional approaches [13, 14, 15, 16, 17, 18].

HCM is defined by LV wall thickness ≥15 mm in adults (or *z* >2 in children), not explained by loading conditions.

**Key Features (**Table 5):

- Asymmetric septal hypertrophy (septal/posterior wall ratio >1.3).
- Systolic anterior motion (SAM) of the mitral valve.
- LVOT obstruction (provokable gradient ≥50 mmHg).
- MR due to SAM: Mitral regurgitation (MR) in hypertrophic cardiomyopathy (HCM) is often caused by systolic anterior motion (SAM) of the mitral valve. During systole, the hypertrophied septum and altered mitral valve geometry cause the anterior leaflet to be drawn toward the outflow tract, resulting in dynamic left ventricular outflow tract (LVOT) obstruction. This abnormal motion leads to incomplete coaptation of the mitral leaflets and posteriorly directed MR. The severity of MR correlates with the degree of SAM and LVOT gradient, contributing to symptoms such as dyspnea and fatigue in affected patients.
- Hyperdynamic LV function: The left ventricular (LV) function often appears hyperdynamic due to the reduced end‐systolic volume and preserved or even enhanced contractility. Despite the presence of diastolic dysfunction and impaired filling, the thickened myocardium generates vigorous systolic contraction, leading to a reduced LV cavity and an ejection fraction that is typically normal or supranormal.
- Diastolic dysfunction: Diastolic dysfunction is a hallmark of hypertrophic cardiomyopathy (HCM) and plays a central role in the pathophysiology of the disease. The hypertrophied, stiff left ventricular (LV) myocardium impairs relaxation and reduces ventricular compliance, leading to elevated filling pressures and impaired ventricular filling during diastole.

**TABLE 5:** Echocardiographic diagnostic criteria for hypertrophic cardiomyopathy.

Criterion	Measurement/Feature	Diagnostic threshold	Notes
Maximal LV Wall Thickness	Usually septum	≥**15 mm** (in adults)	Most important diagnostic criterion; ≥13 mm may be significant in first‐degree relatives
Asymmetric Septal Hypertrophy (ASH)	Septum/posterior wall ratio	≥**1.3–1.5**	ASH pattern is the most common
LV Cavity Size	End‐diastolic dimension	Normal or reduced	Small LV cavity despite hypertrophy
Systolic Function (LVEF)	Ejection fraction	Usually **normal or hyperdynamic (>65%)**	Normal EF despite impaired relaxation
LV Outflow Tract (LVOT) Gradient	Resting or with provocation	≥**30 mmHg** (significant), ≥**50 mmHg** (severe)	Measured at rest and/or with Valsalva or exercise
Systolic Anterior Motion (SAM) of Mitral Valve	Anterior mitral leaflet in systole	Present	Seen in obstructive HCM (HOCM); contributes to LVOT obstruction
Diastolic Function	Doppler indices (E/A, E/e’, decel time)	Impaired relaxation pattern	Diastolic dysfunction is common in HCM
Mitral Regurgitation	Color Doppler	Often eccentric jet	Due to SAM and mitral‐septal contact
Apical Hypertrophy	Apical wall thickness	≥**15 mm**	“Spade‐like” LV cavity on apical views or contrast echo
Myocardial Strain (optional)	Global longitudinal strain (GLS)	Reduced in hypertrophied regions	May detect early disease in genotype‐positive individuals

### 2.3 Restrictive Cardiomyopathy (RCM)

**FIGURE 7:**
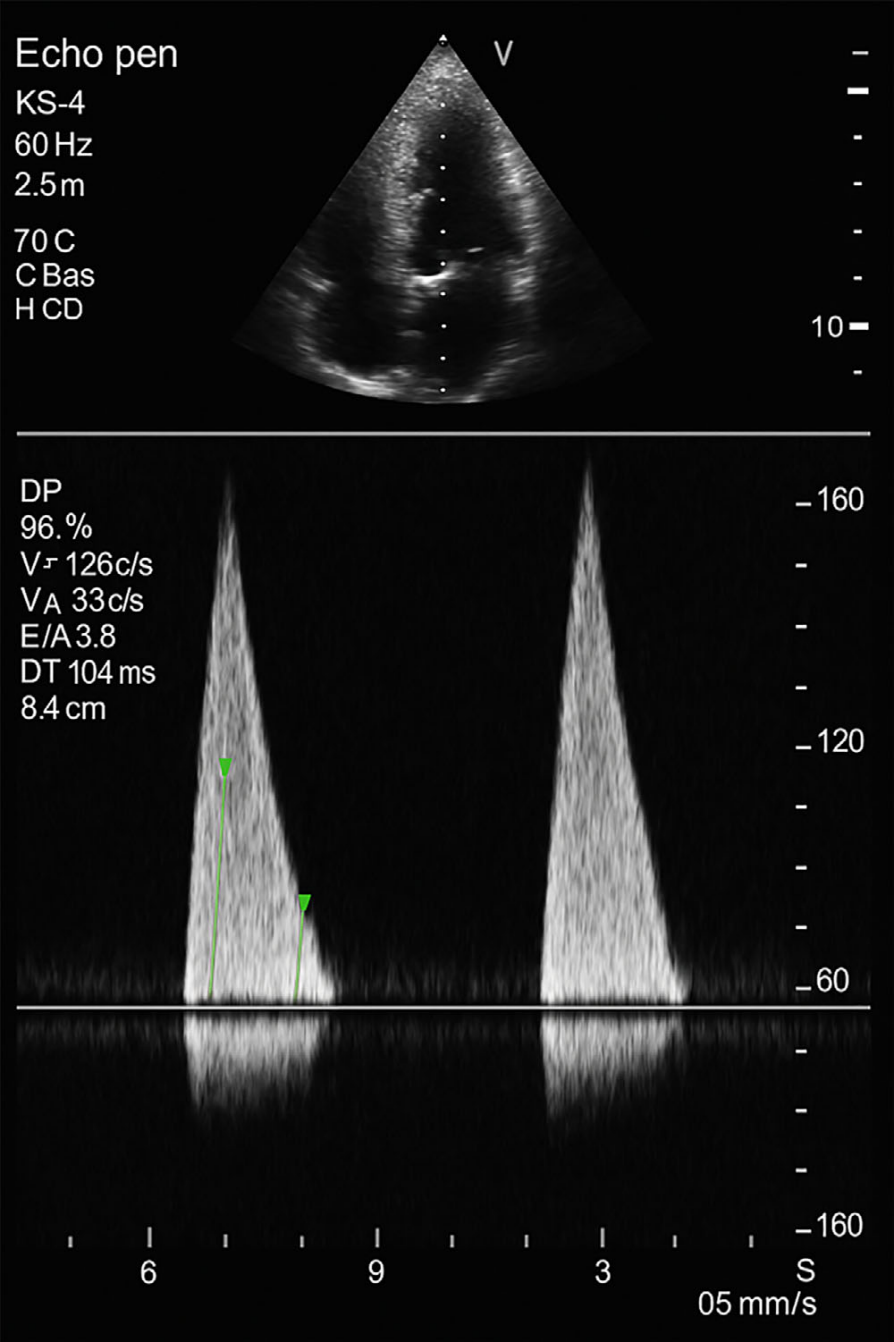
Doppler echocardiographic findings consistent with restrictive cardiomyopathy: markedly elevated early diastolic (E) velocity (126 cm/s), a diminished atrial (A) velocity (33 cm/s), an E/A ratio of 3.8, and a shortened deceleration time (DT) of 104 ms.

**FIGURE 8:**
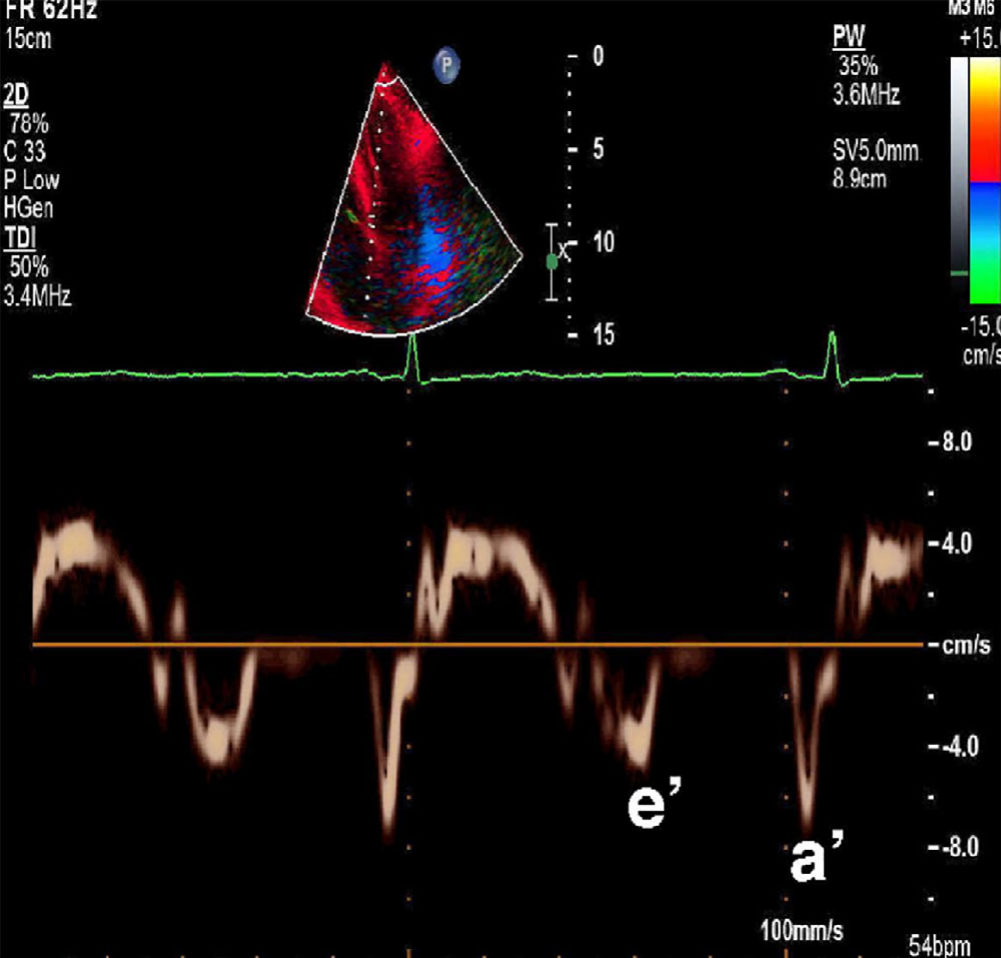
Tissue Doppler imaging (TDI) of the mitral annulus in restrictive cardiomyopathy. The spectral TDI tracing demonstrates a markedly reduced early diastolic mitral annular velocity (e′) and a relatively preserved atrial velocity (a′), consistent with impaired myocardial relaxation. The calculated E/e′ ratio is elevated (18), indicating elevated left ventricular filling pressures, a hallmark of restrictive physiology.

Echocardiography is a fundamental diagnostic modality in the evaluation of **restrictive cardiomyopathy (RCM)**, a rare but severe myocardial disorder characterized by **impaired ventricular filling due to increased myocardial stiffness**, with preserved or near‐normal systolic function, particularly in the early stages. The hallmark echocardiographic feature of RCM is **biatrial enlargement** resulting from chronically elevated filling pressures, often out of proportion to the degree of ventricular hypertrophy or dysfunction. The **ventricular walls are typically non‐dilated and non‐hypertrophied**, distinguishing RCM from hypertrophic and dilated cardiomyopathies, although wall thickening may occur in infiltrative forms such as **amyloidosis**. Doppler echocardiography plays a crucial role in identifying abnormal **diastolic filling patterns**, including a **restrictive transmitral inflow pattern**—marked by a tall E wave (Figure 7), diminished A wave, shortened deceleration time, and reduced E/A variability with respiration—indicative of **elevated left ventricular end‐diastolic pressures**. Tissue Doppler imaging (TDI) further refines diastolic assessment by showing **reduced mitral annular e′ velocities** and **elevated E/e′ ratios (**Figure 8), correlating with elevated left atrial pressure. Infiltrative etiologies like amyloidosis may exhibit characteristic findings such as **increased myocardial echogenicity**, **thickened valves**, and a “sparkling” or “granular” appearance of the myocardium. Strain imaging, especially global longitudinal strain (GLS), may reveal **apical sparing**, a finding highly suggestive of cardiac amyloidosis. Echocardiography is also essential in ruling out **constrictive pericarditis**, which can mimic RCM clinically and hemodynamically. Key differentiating features include **ventricular interdependence**, a **septal bounce**, annulus reversus (**reversal of the normal pattern of early diastolic (e′) tissue Doppler velocities** between the lateral and medial (septal) mitral annulus), and **respiratory variation in mitral inflow velocities** in constrictive pericarditis, all of which are typically absent in RCM (Table 6). In advanced cases of RCM, right ventricular involvement may occur, often demonstrated by reduced tricuspid annular plane systolic excursion (TAPSE) and elevated right atrial pressures. Echocardiography, supplemented by contrast studies or transesophageal echocardiography, when necessary, is indispensable for serial monitoring of disease progression and for assessing the response to treatment, particularly in patients being evaluated for advanced therapies such as heart transplantation [19, 20, 21, 22, 23].

**TABLE 6:** Echocardiographic differences between constrictive pericarditis (CP) and restrictive cardiomyopathy (RCM).

Feature	Constrictive pericarditis (CP)	Restrictive cardiomyopathy (RCM)
Pericardial thickness	Often increased (>2 mm)	Normal
Respiratory variation in mitral/tricuspid inflow	Marked (>25% mitral, >40% tricuspid)	Minimal or absent
Septal motion	Septal bounce or shudder (early diastolic notching)	Normal or reduced
Tissue Doppler e′ velocity (medial)	Preserved or elevated (medial e′ >8 cm/s)	Reduced (medial e′ <8 cm/s)
Annulus reversus	Present (medial e′ > lateral e′)	Absent (lateral e′ > medial e′)
Hepatic vein flow	Prominent expiratory diastolic reversal	Blunted or minimal variation
Left atrial size	Mild to moderate enlargement	Marked enlargement
Pericardial effusion	May be present	Typically absent
Inferior vena cava (IVC)	Dilated with reduced inspiratory collapse	Dilated with reduced collapse (less respiratory variation)

Defined by impaired diastolic filling and preserved or mildly reduced systolic function.

**FIGURE 9:**
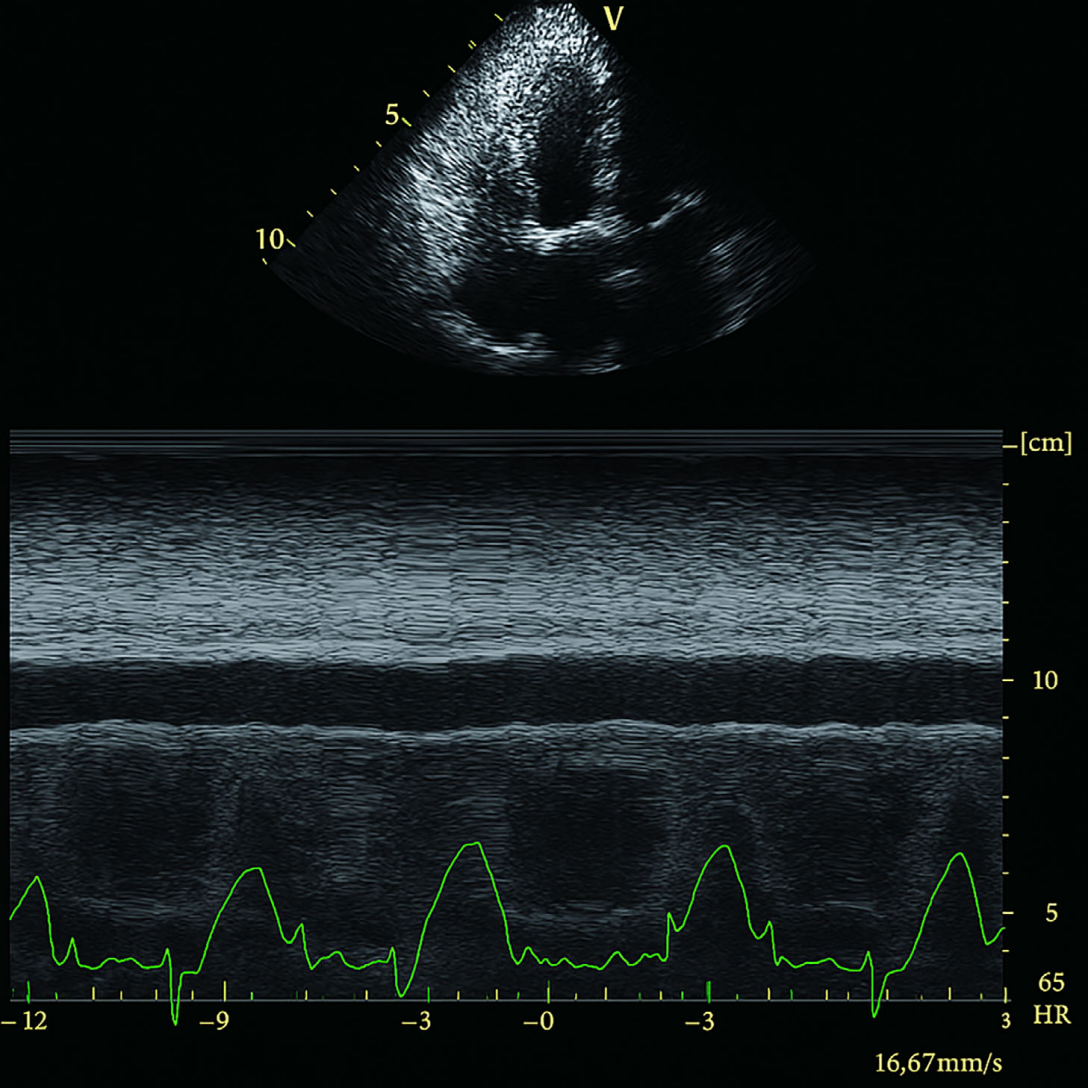
M‐mode echocardiographic image demonstrating dilated inferior vena cava (IVC) with blunted respiratory variation in restrictive cardiomyopathy. The top panel shows a 2D subcostal view of the IVC entering the right atrium. The lower M‐mode tracing illustrates persistently dilated IVC diameter with minimal inspiratory collapse, as shown by the near‐parallel motion of the IVC walls. This finding is consistent with elevated right atrial pressures and impaired ventricular compliance, typical of restrictive physiology.

**Echocardiographic Findings (**Table 7):

- Biatrial enlargement
- Normal wall thickness
- High E/A ratio (>2), short deceleration time (<160 ms)
- Reduced e′ velocity, elevated E/e′ (>15)
- Dilated IVC with reduced respiratory variation (Figure 9)

**TABLE 7:** Echocardiographic diagnostic criteria for restrictive cardiomyopathy.

Criterion	Measurement/Feature	Typical finding in RCM	Notes
Left Ventricular Wall Thickness	Septum and posterior wall	Normal or mildly increased	Increased in infiltrative types (e.g., amyloidosis)
Left Ventricular Cavity Size	End‐diastolic dimension	Normal	Helps distinguish from dilated cardiomyopathy
Systolic Function (LVEF)	Ejection fraction	Normal or mildly reduced	LVEF often preserved until late
Left Atrial Size	LA diameter or volume	**Markedly enlarged**	Due to chronic diastolic dysfunction
Right Atrial Size	RA area or diameter	Often enlarged	Biatrial enlargement is characteristic
Diastolic Function (Doppler)	Mitral inflow (E/A ratio, decel time), TDI e′	**Restrictive filling**: E/A > 2, decel time < 160 ms, **reduced e′**	Most critical finding; indicates impaired relaxation and high filling pressures
Tissue Doppler Imaging (TDI)	Mitral annulus e′ velocity	**e′ < 8 cm/s**, **E/e′ > 15**	Reduced e′ helps differentiate RCM from constriction
Pulmonary Vein Doppler	Systolic/diastolic flow reversal	Blunted systolic flow, atrial reversal >35 cm/s	Suggests elevated LA pressure
Inferior Vena Cava (IVC)	Diameter and collapsibility	Dilated, reduced inspiratory collapse	Reflects elevated right atrial pressure
Pericardium	Pericardial thickness/motion	Normal	Helps differentiate from constrictive pericarditis
Myocardial Texture	Speckle pattern, echogenicity	“Granular sparkling” in amyloidosis	Non‐specific; supports infiltrative etiology
Strain Imaging (GLS)	Global longitudinal strain	Reduced, **apical sparing in amyloidosis**	“Cherry‐on‐top” pattern is classic for cardiac amyloidosis

### 2.4 Arrhythmogenic Right Ventricular Cardiomyopathy (ARVC)

**FIGURE 10:**
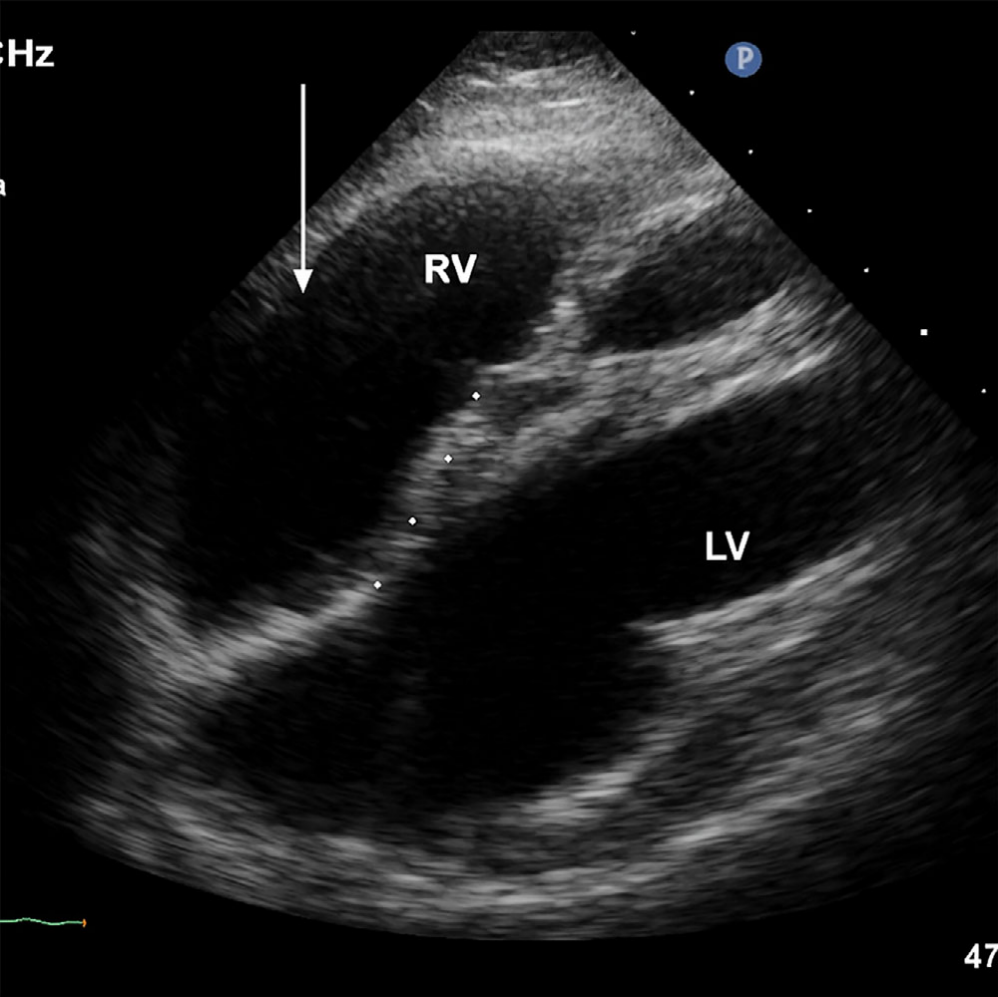
A subcostal echocardiographic image suggestive of arrhythmogenic right ventricular cardiomyopathy (ARVC). This parasternal long‐axis view demonstrates a dilated and hypokinetic right ventricle (RV) with irregular endocardial contours, while the left ventricle (LV) appears structurally normal. The arrow highlights regional RV wall thinning and dyskinesia, which are characteristic findings in ARVC. This structural abnormality, combined with clinical and ECG criteria, supports the diagnosis of ARVC.

Echocardiography is an essential imaging modality in the initial evaluation and longitudinal monitoring of patients with **arrhythmogenic right ventricular cardiomyopathy (ARVC)**, a genetically mediated myocardial disease characterized by **fibrofatty replacement of the right ventricular (RV) myocardium**, leading to ventricular arrhythmias and an increased risk of sudden cardiac death, especially in young individuals and athletes. Although echocardiography may lack sensitivity in detecting early or localized disease compared to cardiac MRI, it remains a widely accessible and non‐invasive tool that contributes critical diagnostic and prognostic information. Classic echocardiographic features of ARVC include **RV dilation**, **reduced RV systolic function**, **regional wall motion abnormalities** (such as hypokinesia, akinesia, or dyskinesia), and **focal aneurysms**, particularly of the **RV outflow tract (RVOT), apex, and inferior wall (**Figure 10). M‐mode and 2D imaging can reveal RV wall thinning and aneurysmal bulging, while Doppler echocardiography may assess tricuspid regurgitation and estimate pulmonary pressures. RV fractional area change (FAC), tricuspid annular plane systolic excursion (TAPSE), and tissue Doppler imaging (TDI) of the tricuspid annulus are often employed to quantify RV systolic function, though their sensitivity may be limited in early disease stages. The 2010 **Task Force Criteria** for the diagnosis of ARVC include specific echocardiographic parameters, such as RV end‐diastolic diameter and regional motion abnormalities, which must be interpreted in the context of other clinical, electrocardiographic, and genetic findings. In addition to evaluating the RV, echocardiography may detect **left ventricular (LV) involvement**, which is now recognized in advanced disease and in certain genetic variants (e.g., desmoplakin mutations), manifesting as subepicardial or midmyocardial fibrosis, wall motion abnormalities, or LV systolic dysfunction. Contrast echocardiography can enhance visualization of endocardial borders in technically difficult cases, while three‐dimensional echocardiography may improve assessment of RV volume and function. Though cardiac MRI remains the gold standard for tissue characterization and detailed RV evaluation, echocardiography continues to be indispensable in screening at‐risk relatives, guiding follow‐up, and monitoring for progressive structural changes in established ARVC [24, 25, 26, 27, 28, 29].

Characterized by Fibrofatty RV Replacement and Arrhythmias.

**Key Features (**Table 8):

- RV dilation, wall motion abnormalities
- RV aneurysm, FAC <33%, TAPSE <17 mm
- RVOT dilation (>32 mm PLAX, >36 mm PSAX)
- Trabecular pattern changes, hyperechogenicity

**TABLE 8:** Echocardiographic diagnostic criteria for arrhythmogenic right ventricular cardiomyopathy (ARVC).

Criterion	Measurement/Feature	Major criteria	Minor criteria	Notes
Regional RV Wall Motion Abnormalities	RV free wall (focused apical/subcostal/TAPSE views)	Akinesia, dyskinesia, or aneurysm	Same as major	Wall motion abnormalities are central to diagnosis
Right Ventricular Outflow Tract (RVOT) Diameter	Parasternal long axis (PLAX)	>32 mm (PLAX)	29–32 mm (PLAX)	Indexed >19 mm/m^2^ (major), 16–19 mm/m^2^ (minor)
	Parasternal short axis (PSAX)	>36 mm (PSAX)	32–36 mm (PSAX)	
Right Ventricular Fractional Area Change (FAC)	% change in area	<33%	33%–40%	Indicates reduced RV systolic function
Tricuspid Annular Plane Systolic Excursion (TAPSE)	M‐mode of the lateral tricuspid annulus	Often reduced (<17 mm)	—	Not part of Task Force criteria but supportive
Right Atrial and RV Size	Apical 4‐chamber RV‐focused view	Enlarged RV or RA	Mild enlargement	Not part of formal criteria, but supports diagnosis
LV Involvement (if present)	LV strain, wall motion	LV dysfunction or strain abnormalities	—	Seen in biventricular or left‐dominant forms
Tissue Doppler and Strain Imaging	RV free wall longitudinal strain	Reduced (>–20%)	—	Advanced echo feature to detect early dysfunction
Echogenicity	RV myocardium appearance	Increased echogenicity, trabeculations	—	Suggestive but non‐specific
Contrast Echocardiography	RV cavity enhancement	Enhances the detection of aneurysms	—	Used if conventional echo is suboptimal

Diagnosis of ARVC requires a combination of major and minor criteria from different categories: imaging, ECG, arrhythmia, histopathology, genetics, and family history.

Echocardiography is first‐line, but cardiac MRI is more sensitive for tissue characterization and detection of subtle RV abnormalities.

Always integrate clinical context and arrhythmic findings when interpreting the echo in suspected ARVC.

**LV Involvement**: Seen in advanced stages; includes wall motion abnormalities and strain reduction.

### 2.5 Left Ventricular Non‐Compaction (LVNC)

**FIGURE 11:**
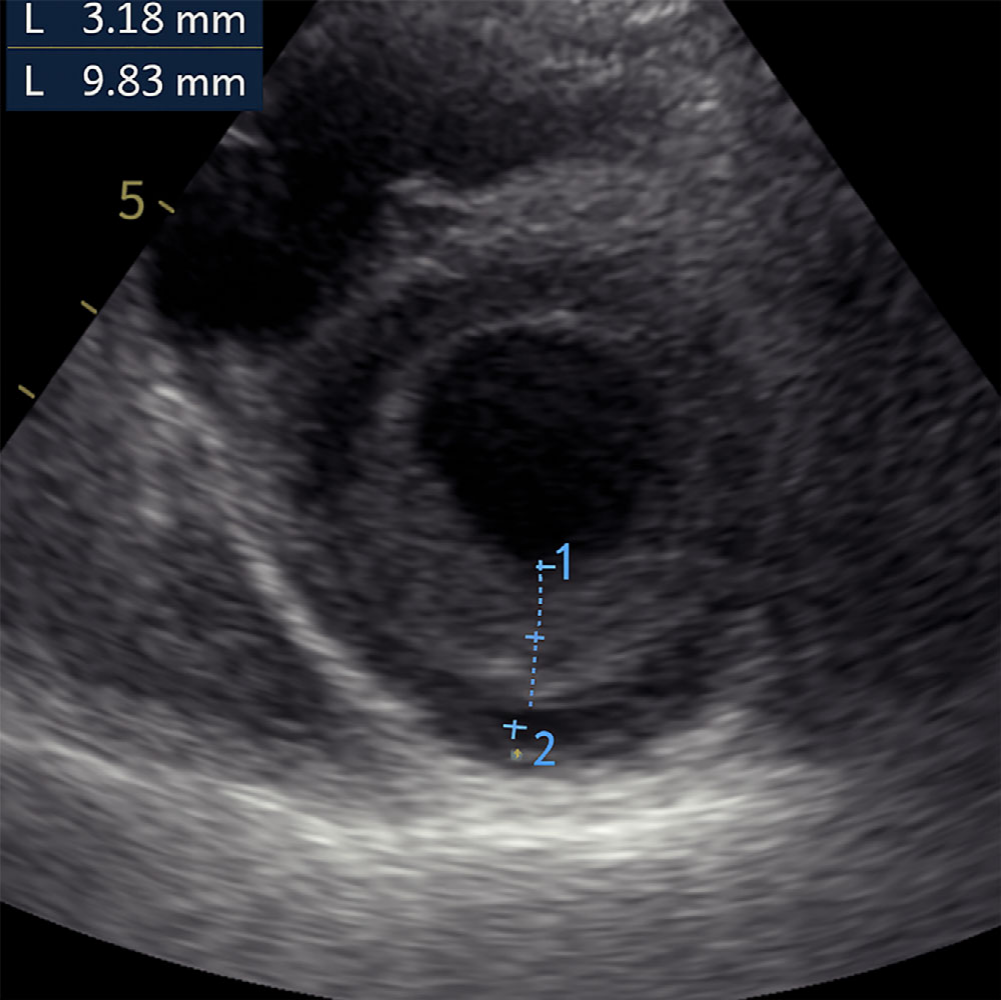
Apical short axis view of LV showing >2 ratio of non‐compacted to compact myocardium. This finding (Jennie criteria) should be added to other criteria to diagnose LVNC cardiomyopathy.

Echocardiography is a primary imaging modality for the diagnosis and assessment of **left ventricular non‐compaction cardiomyopathy (LVNC)**, a rare myocardial disorder characterized by **prominent trabeculations** and **deep intertrabecular recesses** resulting from an arrest in normal embryonic endomyocardial compaction. The condition most commonly affects the **apical and mid‐ventricular segments of the left ventricle**, although biventricular or isolated right ventricular involvement can also occur. On two‐dimensional transthoracic echocardiography, key diagnostic features include a **two‐layered myocardial structure** with a thin, compacted epicardial layer and a markedly thicker non‐compacted endocardial layer. The **Jenni criteria**, widely used in echocardiographic diagnosis, define LVNC based on a non‐compacted to compacted (NC/C) myocardial ratio greater than **2:1 during systole (**Figure 11), most notably in the parasternal short‐axis or apical views. Color Doppler imaging further supports the diagnosis by demonstrating **blood flow within the deep intertrabecular recesses**, confirming their communication with the ventricular cavity. Three‐dimensional echocardiography and contrast‐enhanced studies can enhance the delineation of the trabecular architecture and improve diagnostic accuracy, particularly in technically challenging cases. Functional assessment typically reveals **left ventricular systolic dysfunction**, although diastolic dysfunction and regional wall motion abnormalities may also be present. Importantly, the degree of non‐compaction does not always correlate with functional impairment, and some individuals may remain asymptomatic. Tissue Doppler imaging and speckle‐tracking echocardiography may reveal **subclinical myocardial dysfunction**, even when the global ejection fraction is preserved. LVNC is often associated with **arrhythmias, thromboembolic events, and progressive heart failure**, making risk stratification crucial; echocardiography aids in the identification of intracavitary thrombi and provides a basis for therapy decisions, including anticoagulation, implantable cardioverter‐defibrillators, or advanced heart failure therapies. While **cardiac magnetic resonance imaging (CMR)** offers superior tissue characterization and more sensitive quantification of trabeculation, echocardiography remains a vital, first‐line, and widely available tool in the diagnosis and longitudinal monitoring of LVNC, particularly in resource‐limited settings or as a screening modality for affected family members [30, 31, 32, 33, 34, 35].

**Defined by Prominent Trabeculations and Intertrabecular Recesses**

**Diagnostic Criteria (**Table 9):

- NC/C ratio >2:1 in end‐systole
- Apical/mid‐ventricular involvement
- Color Doppler showing flow in recesses
- Possible systolic dysfunction

**TABLE 9:** Echocardiographic diagnostic criteria for left ventricular noncompaction cardiomyopathy (LVNC).

Criterion	Measurement/Feature	Diagnostic threshold	Notes
Two‐layered Myocardium	Compact (C) + Noncompacted (NC) layers	NC/C ratio > **2.0** at end‐systole (Jenni criteria)	Most widely used echocardiographic criterion
Location of Prominent Trabeculations	Apical, mid‐lateral, mid‐inferior walls	Typically in ≥2–3 segments according to the author	Basal involvement is atypical and raises suspicion of artifact
Color Doppler Flow	Between trabeculae	Demonstrable deep intertrabecular recesses perfused from the LV cavity	Confirms communication with the ventricular cavity
Wall Motion Abnormalities	Regional or global	Often present	LV systolic dysfunction is common but not required
LV Size	LV end‐diastolic diameter	Normal or dilated	LV dilation is seen in advanced cases
LV Ejection Fraction (EF)	Systolic function	Normal to severely reduced	EF may be preserved early, and reduced with disease progression
Noncompacted to Compacted Ratio (Chin Criteria)	Measured at end‐diastole	X/Y < **0.5** (X = distance from epicardium to trough; Y = epicardium to peak)	Less commonly used than Jenni
Strain Imaging (GLS)	Global longitudinal strain	Abnormal in the affected segments	May detect subclinical dysfunction
Contrast Echocardiography	LV opacification	Enhances visualization of trabeculations	Helpful when image quality is suboptimal

Diagnosis should be based on a comprehensive evaluation, not imaging alone.

LVNC can be physiologic in athletes, pregnant women, or in children—clinical correlation is essential.

Cardiac MRI offers higher resolution and is often used for confirmation when echo findings are inconclusive.

Major LVNC diagnostic criteria used in practice are summarized in Table 10.

**TABLE 10:** LVNC key diagnostic criteria used in practice [36, 37, 39].

Author	Modality	Phase	Threshold
Jenni et al.	Echo	End‐systole	NC/C > **2.0**
Chin et al.	Echo	End‐diastole	X/Y < **0.5**
Stollberger et al.	Echo	NA	≥**3 trabeculations** moving synchronously with myocardium

## 3 Prognostic and Therapeutic Implications

Understanding the prognostic indicators and therapeutic implications of various cardiomyopathies is essential for risk stratification, clinical decision‐making, and individualized patient management. Echocardiography not only aids in diagnosis but also plays a pivotal role in monitoring disease progression and guiding treatment strategies. Table 11 below summarizes key echocardiographic parameters associated with adverse outcomes in each cardiomyopathy subtype and outlines their relevance to current therapeutic approaches.

**TABLE 11:** Echocardiographic prognostic and therapeutic implications of major cardiomyopathy subtypes.

Cardiomyopathy type	Prognostic indicators	Therapeutic implications
Dilated Cardiomyopathy (DCM)	‐ ↓ LVEF (<35%) ‐ ↑ LVEDD/LVEDVi ‐ ↓ RV function (TAPSE, RV FAC) ‐ ↑ LV mass index ‐ ↑ Mitral regurgitation severity ‐ Reduced GLS	‐ Guideline‐directed medical therapy (GDMT): ACEi/ARB/ARNI, beta‐blockers, MRA ‐ CRT/ICD in patients with reduced EF and wide QRS ‐ Anticoagulation if apical thrombus is present ‐ Heart transplant evaluation in refractory cases ‐ Serial echo for remodeling and therapy response
Hypertrophic Cardiomyopathy (HCM)	‐ Maximal wall thickness ≥30 mm ‐ LVOT gradient ≥50 mmHg ‐ ↓ GLS in hypertrophied segments ‐ LA enlargement ‐ Presence of apical aneurysm ‐ Family history of SCD or prior VT/VF	‐ Risk stratification for ICD implantation (e.g., ESC SCD Risk Calculator) ‐ Beta‐blockers or CCBs for symptomatic obstruction ‐ Septal myectomy or alcohol septal ablation if refractory ‐ Anticoagulation in atrial fibrillation ‐ Echo‐guided follow‐up for obstruction, aneurysm, or disease progression
Restrictive Cardiomyopathy (RCM)	‐ Restrictive filling pattern (E/A >2, ↓ DT) ‐ ↑ E/e′ ratio (>15) ‐ ↓ GLS, especially apical sparing (amyloidosis) ‐ Biatrial enlargement ‐ RV dysfunction ‐ Elevated pulmonary pressures	‐ Diuretics for volume management ‐ Anticoagulation in atrial fibrillation or LA enlargement ‐ Therapy tailored to etiology (e.g., tafamidis for ATTR amyloidosis) ‐ Echo helps differentiate from constrictive pericarditis ‐ Consider heart transplant in progressive cases
Arrhythmogenic Right Ventricular Cardiomyopathy (ARVC)	‐ RV wall motion abnormalities (akinesia/dyskinesia) ‐ RV dilation and ↓ FAC (<33%) ‐ ↓ TAPSE ‐ LV involvement ‐ Family history of SCD ‐ Documented ventricular arrhythmias	‐ Lifestyle modification: avoid competitive athletics ‐ Beta‐blockers or antiarrhythmics (e.g., sotalol) ‐ ICD for primary or secondary prevention ‐ Family screening ‐ Serial echo and/or MRI to monitor structural progression
Left Ventricular Non‐Compaction (LVNC)	‐ Reduced EF ‐ ↑ LVEDD ‐ ↓ GLS in non ‐ compacted segments‐ Presence of thrombus ‐ Ventricular arrhythmias or conduction abnormalities	‐ GDMT for systolic dysfunction ‐ Anticoagulation if apical thrombus or aneurysm ‐ ICD in high‐risk patients (low EF, arrhythmias) ‐ Family screening and genetic counseling ‐ Serial echo to track function and thrombus risk

## 4 Limitations of Echocardiography in Cardiomyopathy Diagnosis

Despite its widespread use and diagnostic utility, echocardiography has several inherent limitations in the evaluation of cardiomyopathies. Image acquisition and interpretation are highly operator‐dependent, and the diagnostic accuracy can be significantly affected by patient body habitus, acoustic window quality, and sonographer experience. In early or segmental disease—such as arrhythmogenic right ventricular cardiomyopathy (ARVC) or infiltrative forms like cardiac amyloidosis—structural changes may be subtle or focal and therefore difficult to detect with conventional echocardiography. Additionally, echocardiography lacks the ability to directly characterize myocardial tissue, limiting its sensitivity in differentiating between fibrotic, fatty, or infiltrative pathologies. These constraints underscore the importance of multimodal imaging approaches and highlight the complementary role of cardiac magnetic resonance imaging (CMR) or computed tomography (CT) in select cases, particularly when echocardiographic findings are inconclusive or discordant with clinical suspicion.

## 5 Conclusion

Echocardiography remains an essential and first‐line imaging modality in the comprehensive evaluation and management of cardiomyopathies. Table 12 summarizes the key echocardiographic features that distinguish the five major cardiomyopathy subtypes, including chamber morphology, functional impairments, valve abnormalities, and advanced diagnostic criteria such as strain analysis and Doppler patterns. A systematic approach that integrates quantitative chamber measurements, functional assessment, and age‐ or size‐specific normative data—particularly in both adult and pediatric populations—significantly enhances diagnostic accuracy and supports informed clinical decision‐making. Advanced echocardiographic techniques, including tissue Doppler imaging, speckle‐tracking strain analysis, and contrast‐enhanced imaging, further improve sensitivity for detecting early or subtle myocardial dysfunction and allow for a more nuanced understanding of cardiac mechanics.

**TABLE 12:** Overview of differentiating echocardiographic features across cardiomyopathy subtypes.

Type	Key echocardiographic features
Dilated Cardiomyopathy (DCM)	‐ LV and/or RV dilation ‐ EF < 40% (systolic dysfunction) ‐ Global hypokinesis ‐ Functional MR/TR due to annular dilation ‐ Possible apical thrombus ‐ ↓ TAPSE and RV FAC if RV involved ‐ Elevated LVEDD, LVEDVi, LV Mass Index ‐ Relative wall thickness (RWT) < 0.42
Hypertrophic Cardiomyopathy (HCM)	‐ LV wall thickness ≥15 mm (or *z* >2 in children) ‐ Asymmetric septal hypertrophy (ASH), septum/posterior wall ratio ≥1.3‐ Systolic anterior motion (SAM) of the mitral valve ‐ Dynamic LVOT obstruction (≥30 mmHg, ≥50 mmHg severe) ‐ Eccentric MR from SAM ‐ Hyperdynamic LV (EF >70%) ‐ Diastolic dysfunction (↓ E/A ratio, ↓ e′) ‐ Apical aneurysms possible
Restrictive Cardiomyopathy (RCM)	‐ Biatrial enlargement‐ Normal or mildly thickened ventricular walls ‐ Preserved or mildly ↓ EF‐ Restrictive diastolic filling pattern (E/A >2, decel time <160 ms) ‐ ↓ e′ and ↑ E/e′ ratio (>15) ‐ Dilated IVC with ↓ respiratory variation ‐ No septal bounce (differentiates from constrictive pericarditis) ‐ “Granular” myocardium in amyloidosis
Arrhythmogenic Right Ventricular Cardiomyopathy (ARVC)	‐ RV dilation and wall thinning ‐ Regional wall motion abnormalities: akinesia, dyskinesia, or aneurysm ‐ ↓ RV FAC (<33%), ↓ TAPSE (<17 mm) ‐ ↑ RVOT diameter (>32 mm PLAX, >36 mm PSAX) ‐ Echogenicity changes and trabeculation ‐ LV involvement in advanced stages ‐ Use of contrast echo to detect RV aneurysms
Left Ventricular Non‐Compaction (LVNC)	‐ Prominent trabeculations with deep recesses ‐ NC/C ratio >2:1 (Jenni criteria, end‐systole) ‐ Affects apical/mid‐ventricular walls ‐ Color Doppler: flow in intertrabecular recesses ‐ Regional or global wall motion abnormalities ‐ EF: Normal to ↓‐ ≥3 trabeculations beyond papillary muscles (Chin criteria) ‐ Abnormal GLS in affected segments

However, in cases where echocardiographic findings are limited by poor acoustic windows or lack specificity—such as in infiltrative, arrhythmogenic, or apical pathologies—additional modalities such as **cardiac magnetic resonance (CMR)** and **cardiac computed tomography (CT)** play a complementary role. These techniques offer superior tissue characterization, scar detection, and detailed anatomical visualization. As imaging technology continues to evolve, a multimodal approach incorporating echocardiography, CMR, and CT will remain central to the personalized, evidence‐based care of patients with cardiomyopathy.
